# FairBase: a comprehensive database of fungal A-to-I RNA editing

**DOI:** 10.1093/database/baz018

**Published:** 2019-02-19

**Authors:** Jinding Liu, Dongbo Wang, Yinna Su, Kun Lang, Rongjing Duan, YuFeng Wu, Fei Ma, Shuiqing Huang

**Affiliations:** 1 College of Information Science and Technology, Nanjing Agricultural University, Nanjing, China; 2 Research Center for Correlation of Domain Knowledge, Nanjing Agricultural University, Nanjing, China; 3 Bioinformatics center, Nanjing Agricultural University, Nanjing, China; 4 College of Life Science, Nanjing Normal University, Nanjing, China; 5 Laboratory for Comparative Genomics and Bioinformatics, Nanjing Normal University, Nanjing, China

## Abstract

Frequent A-to-I RNA editing has recently been identified in fungi despite the absence of recognizable homologues of metazoan ADARs (“Adenosine Deaminases Acting on RNA”). In particular, there is emerging evidence showing that A-to-I editing is involved in sexual reproduction of filamentous fungi. Here, we report on the creation of FairBase — a fungal A-to-I RNA editing database that provides a platform for deep exploration of fungal RNA editing to relevant academic communities. This database includes a comprehensive collection of A-to-I editing sites in six filamentous fungal species, together with extensive annotations for each editing site. In FairBase, users can conveniently search editing sites and obtain editing levels for each editing site in various RNA-seq samples. In addition, the pathways involving RNA editing are built in FairBase to help users understand the functions of RNA editing. Furthermore, each fungal species has a genome browser (JBrowse) that allows users to explore A-to-I editing in a genomic context. FairBase is the first fungal RNA editing database.

## Introduction

RNA editing is a class of co−/post-transcriptional modification that can alter hereditary information in the genome by changing the nucleotide sequence of RNA molecules (1–3). A-to-I RNA editing catalyzed by members of the adenosine deaminase acting on RNA (ADAR) family of enzymes is the main type of RNA editing in animals; this editing can expand the transcriptome and proteome (4). Studies have revealed that A-to-I editing can be involved in the regulation protein recoding, alternative splicing, RNA interference (RNAi) and microRNA expression (5–8). Moreover, A-to-I editing can also be involved in the progressions of many diseases including cancer, neurological deterioration, and metabolic disorders (9–13). In recent years, the advent of high-throughput RNA sequencing (RNA-seq) has enabled transcriptome-wide identification of RNA editing sites and has greatly accelerated the discovery of A-to-I editing sites. The boom in identification of A-to-I editing site has necessitated the development of RNA editing databases to help elucidate biological functions of RNA editing. Some RNA editing databases have been created and are used for large-scale collection and annotation of RNA editing for animals. For example, DARNED is the earliest A-to-I RNA editing database, including editing sites for human, mouse, and fruit fly (14). RADAR not only expanded the number of editing sites but also incorporated editing levels in RNA-seq samples and conservation of editing to other species (15). Recently, REDIportal was designed to embed RADAR database and represents the first editing resource designed to answer functional questions, enabling the inspection and browsing of editing levels in a variety of human samples, tissues and body sites (16).

In fungi, A-to-I RNA editing is usually thought to be scarce due to the lack of orthologs of animal ADARs. Until now, only a small number of large-scale RNA editing discovery studies and very few functional experiments for A-to-I RNA editing have been reported in fungi (17–21). Based on RNA-seq data, thousands of RNA editing sites have been identified in *Ganoderma lucidum* (17) and *Fomitopsis pinicola* (21), but no preference for A-to-I over other forms of editing was found, and the functional significance of A-to-I RNA editing was not studied. In *Fusarium graminearum,* more than 26000 stage-specific A-to-I RNA editing sites were identified during sexual development (18). Furthermore, the editing of RNA encoding a protein kinase PUK1 (perithecium unique kinase) was experimentally shown to play an important role in ascospore formation and release (18). Similarly, more than 40000 stage-specific A-to-I RNA editing sites were identified at different sexual stages in *Neurospora crassa* (19). The comprehensive analyses of A-to-I editing in *N. crassa* showed A-to-I editing was generally adaptive, and might to be functionally related to repeat induced point mutation and meiotic silencing by unpaired DNA (19, 22). A-to-I RNA editing during sexual development also occurred in *Sordaria macrospora* and *Pyronema confluens,* two distantly related filamentous ascomycetes, suggesting that stage-specific A-to-I editing might be an evolutionary conserved feature during sexual development in filamentous ascomycetes (20).

Despite recent advances in fungal RNA editing, the exact biological role of A-to-I RNA editing, and the adenosine deamination mechanism have not been elucidated in fungi. Systematically gathering and sorting the resources of RNA editing could provide the reference information when further probing the functions and molecular mechanisms of RNA editing, even when performing cross-species functional verification in different species. In this work, using almost all available RNA-seq data of filamentous fungi, we developed FairBase, the first fungal A-to-I editing database. FairBase includes a comprehensive collection of A-to-I editing sites, together with extensive annotations and editing levels for each site. In addition, FairBase provides various query functions and graphical visualization pages to facilitate access to RNA editing data.

**Table 1:** Statistic of A-to-I editing events and sites collected in FairBase

Species	RNA-seq samples	Editing events	Editing sites (Nonsyn%^*^)
*F. graminearum*	12	303279	48508 (60%)
*N. crassa*	11	161197	47346 (50%)
*N. tetrasperma*	5	32355	28492 (53%)
*F. verticillioides*	1	5227	5227 (72%)
*P. omphalodes*	1	8238	5217 (73%)
*S. macrospora*	2	2423	2423 (65%)
Total	32	512719	137213 (56%)

^*^Nonsyn%, Percentage of nonsynonymous editing sites occurred in coding regions.

## Material and methods

### RNA-seq data collection and preparation

Only RNA-seq samples with available reference genome assemblies in the Ensembl Fungi database (23) and available RNA-seq data in the NCBI Sequence Read Archive (SRA) database (24) were collected for identification of A-to-I RNA editing. A total of 6253 raw RNA-seq data in SRA format were downloaded and converted into FASTQ format. Then, Trimmomatic (25) was used to cut sequencing adapters and low quality sequences from the 3′ and 5′ end until the base quality score was at least 10. To obtain high-quality clean data, reads with an overall mean Phred-scaled value less than 20 were discarded. Clean reads of each RNA-seq sample were aligned to a reference genome using Hisat2 (26) and read alignments were saved in mapped bam files. Only RNA-seq samples with more than 70% mapped reads were used for the identification of RNA editing events.

### A-to-I RNA editing calling

Duplicated reads were removed from mapped bam files using the MarkDuplicates program in the Picard package (https://broadinstitute.github.io/picard). REDItool was used to call RNA variants present in at least five reads with a minimum frequency of 3% and minimum coverage of 10 reads (27). For non-strand-specific RNA-seq data, mismatch type was inferred by gene annotation and RNA variants occurring in the intergenic region were discarded. Sine noncanonical RNA variants tend to be false-positives in animals (28–30), to improve the reliability of A-to-I RNA editing event, RNA-seq samples without significant preference for A-to-I RNA variants also were discarded. Finally, a total of 32 RNA-seq samples were retained, including 7 samples designed for fungal RNA editing studies (18, 19) and 25 newfound RNA-seq samples.

In the newfound RNA-seq samples, there were 13 samples with RNA-seq strains were inconsistent with reference genome strains (Table S1). The single nucleotide polymorphisms (SNPs) reported in previous studies (31, 32) were used to exclude SNPs at the transcript level in six *F. graminearum* RNA-seq samples with inconsistent reference genome strains. For the remaining seven RNA-seq samples, the whole genome shotgun sequencing (WGS) data from RNA-seq strains were aligned to reference genomes to add genomic support for the exclusion of SNPs at the transcript level (Table S1). Furthermore, A-to-I RNA variants with extreme degree of variance (>90%), which were most likely to be genomic variants, were excluded from the 13 RNA-seq samples.

### Annotation of A-to-I RNA editing sites

The editing events reported in the seven RNA-seq samples from previous studies (18, 19) and the editing events identified in the remaining 25 RNA-seq samples were included in FairBase. The positions of RNA editing events of each fungal species were merged to yield a comprehensive and non-redundant catalogue of editing sites. For each editing site, we curated annotations including genome context, codon change, amino acid change, and edited gene. To support the data retrieval with functions of edited gene, Gene ontology (GO) and Protein family (Pfam) function annotations were predicted using InterProScan (33). Furthermore, the pathways involving RNA editing were built using Kyoto Encyclopedia of Genes and Genomes (KEGG) database (34).

### Implementation of FairBase

We implemented the FairBase database using MySQL as the back-end database, PHP and Perl for the server-side scripting, as well as JavaScript and JQuery plugins as front-end interface. Moreover, the JBrowse genome browser (35) was also embedded in FairBase for users to explore editing sites in genomic context.

## Results

### Database content

Currently, a total of 32 RNA-seq samples with significant preference for A-to-I editing are deposited in FairBase. These RNA-seq samples across six fungi, including *Fusarium graminearum*, *Fusarium verticillioides*, *Neurospora crassa*, *Neurospora tetrasperma, Pyronema omphalodes* and *Sordaria macrospora* and are all related to fungal sexual reproduction (Table S1). In the 32 RNA-seq samples, a total of 512719 A-to-I editing events are detected on 137213 editing sites (Table 1). Of these editing sites, about 70% occur in coding regions (CDSs) and 56% editing sites can result in nonsynonymous recoding, which can result in amino acid change.

### Web Interface

**Figure 1:**
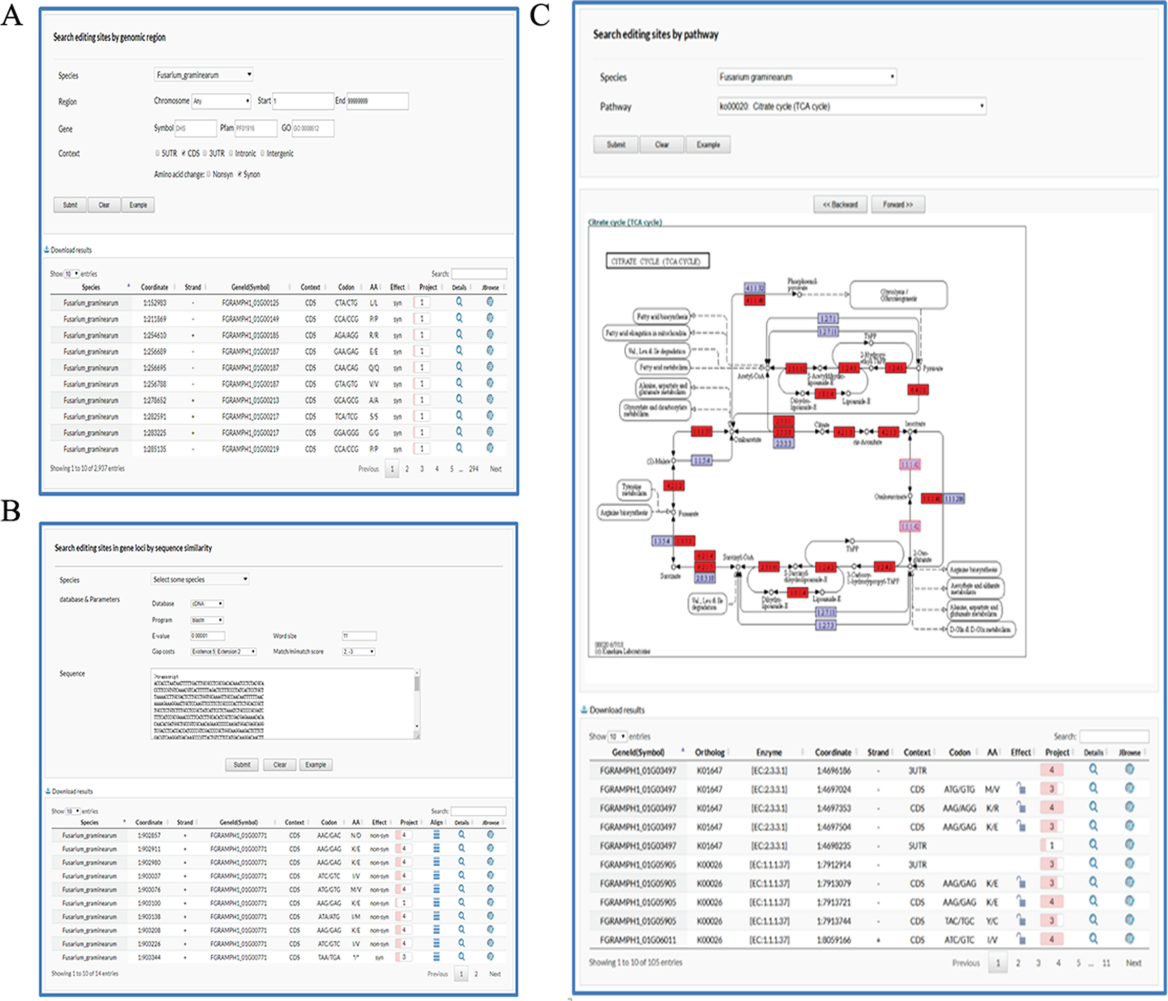
Retrieval of editing sites in FairBase. (A) The search page. Editing sites can be searched by genomic position including species, chromosome, and coordinate. Users can define extra filters to restrict retrieval results to the editing sites of interest. (B) The BLAST page. Users can search editing sites in genes based on the sequence similarity to a user-specified query sequence. (C) The pathway page. Users can search editing sites occurring in a specified pathway, in which the edited genes are tagged with a red background.

**Figure 2:**
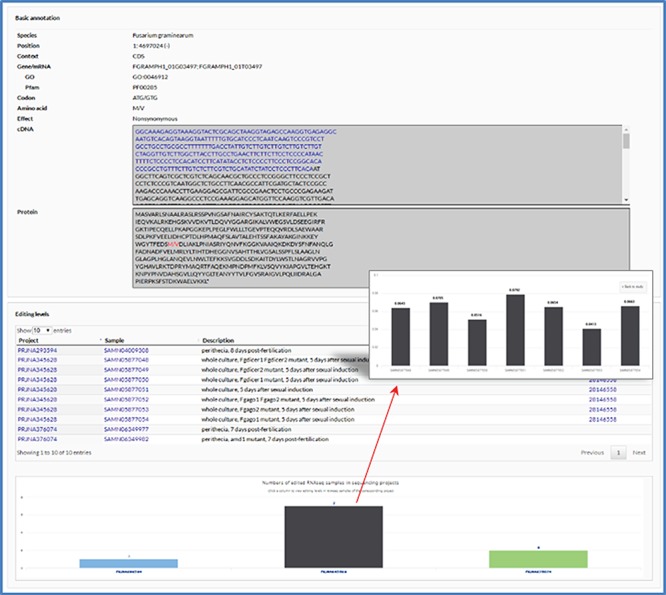
The editing detail page. The editing detail page comprises two sections of information for the editing site, including annotation of editing site and editing levels in RNA-seq samples.

**Figure 3:**
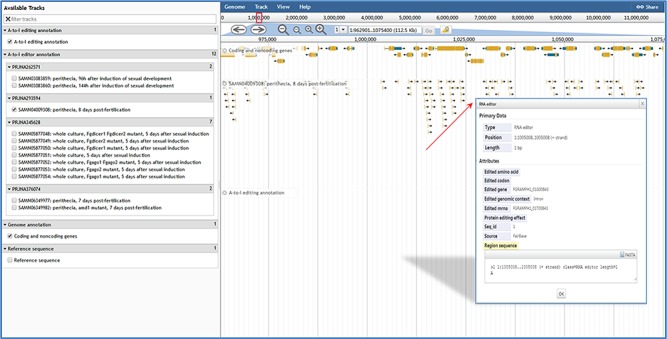
JBrowse in FairBase. Editing sites can be explored in a genomic context using JBrowse. Users can obtain the editing details by clicking on an editing event.

User-friendly web interfaces are designed for users to access the FairBase database. Data retrieval can be achieved in the search, blast and pathway page and the retrieved editing sites are listed in a sortable and downloadable table below (Figure 1). The complete description of an editing site is shown in the editing detail page (Figure 2). All editing sites and editing events can be explored in genome context in the JBrowse genome browser (Figure 3).

In the Search page, editing sites can be searched by genomic position including species, chromosome and coordinate (Figure 1A). Users can define extra filters to restrict retrieval results to the editing sites of interest: (i) Gene, selecting editing sites in edited genes with specified symbol as well as Pfam and GO terms; (ii) Context, selecting editing sites in specified genomic context such as 5’UTR (Untranslated region), CDS (Coding region), 3’UTR, Intronic and Intergenic region; and (iii) AA change, selecting editing sites in CDSs involved in specified amino acid changes, e.g. synonymous and nonsynonymous.

In the blast page, users can search editing sites in genes based on their sequence similarity to a user-specified query sequence (Figure 1B). To meet multiple search purposes, four types of sequence data including DNA, cDNA, CDS and protein sequences of genes are built in FairBase for BLAST search. Users can adjust a BLAST search by word size, match, mismatch, and gapping scores.

In the pathway page, users can search editing sites occurring in a specified pathway (Figure 1C). After users submit a query, the specified pathway is shown below and the edited genes are tagged with red background. The corresponding enzyme identifiers of edited genes also occur in the retrieved results table so as to help users understand RNA editing in pathway context.

In the retrieved results table, at least 10 columns of information are provided to describe an editing site (Figure 1A-C): (i) Genome position; (ii) Editing orientation; (iii) Identifier and Symbol (if any) of edited gene; (iv) Genomic context; (v) Codon change; (vi) Amino acid change; (vii) Editing effect in CDSs, e.g. synonymous and nonsynonymous; (viii) Number of sequencing projects; (ix) Icon with a hyperlink to details page; and (x) Icon with a hyperlink to JBrowse.

The editing detail page contains two sections of information for an editing site including annotation of editing site and editing levels in RNA-seq samples (Figure 2). In the first section, in addition to the information listed in the retrieval results table, relevant sequences are also shown when an editing site occurs in gene loci. In the second section, a table is used to list RNA-seq samples and a column chart is used to show numbers of edited RNA-seq samples in each sequencing project. By clicking on a column, users can switch to another column graph to view the editing levels in RNA-seq samples.

When users submit a query in the browse page or click the JBrowse icon in the retrieval results table, the genome browser JBrowse opens for users to view editing sites in the genomic context (Figure 3). In addition to annotations of genes and A-to-I editing sites, tracks of RNA editing events in RNA-seq samples also are built into JBrowse. By clicking on editing events in these tracks, users can obtain basic annotation information of editing site and editing levels in RNA-seq samples.

## Discussion and future prospects

Many studies have shown the A-to-I RNA editing is involved in various biological processes in animals, while we have very little knowledge about fungal A-to-I RNA editing. To help understand A-to-I RNA editing in fungi, we developed the FairBase database to collect fungal A-to-I RNA editing sites and provide retrieval functions. In FairBase, GO/Pfam functional annotations of edited genes are used to help users quickly lock up editing sites of interest in function-related edited genes. In addition, FairBase provides the BLAST search function, which not only enables the retrieval of editing sites through sequence similarity, but also contributes to the cross-species functional verification between edited genes in FairBase and out of FairBase. Furthermore, KEGG pathways and JBrowse genome browser are built in FairBase for users to explore editing sites in a pathway and genome context. Compared with several animal RNA editing databases (14–16), FairBase collects more species and provides more retrieval functions (Table S2).

It is challenging work to identify RNA editing sites from public available data, because many factors, such as sequencing errors, SNPs among different strains, and other problems can affect the reliability of results. Thus the strict strategies were used in this study. For example, only RNA-seq samples with at least 70% mapped reads can be used for identification of RNA editing events. In addition, in view of the fact that noncanonical RNA edits are found to be false-positives (28–30, 36) and A-to-I RNA editing are enriched in animals RNA-seq samples (37, 38), RNA-seq samples without significant enrichment for A-to-I RNA editing also were discarded in this study. Although thousands of RNA-seq samples were gathered for identification of A-to-I editing sites, ultimately only 32 RNA-seq samples related to sexual reproduction were retained. The results seem to show that A-to-I RNA editing occurs specifically during sexual reproduction. However, whether A-to-I RNA editing is related to other developmental or infection stages still is an open issue (22). It is anticipated that high-throughput sequencing will be continually applied in various fungal studies, including the function and mechanism of RNA editing. With more high-throughput omics data available, we will regularly harvest fungal A-to-I editing data and keep FairBase up-to-date. In addition, the retrieval functions will be further enriched, such as support for the retrieval of editing sites according to codon-change and editing efficiencies and so on. We believe this database and its future updates will be a valuable resource to boost fungal RNA editing researches.

## Supplementary Material

Supplementary Data

## Notes

**Database URL:** bioinfo.njau.edu.cn/fairbase
