# Supplementary material for: FairBase: a comprehensive database of fungal A-to-I RNA editing
Source: Database (Oxford). 2019 Feb 19;2019:baz018. doi: 10.1093/database/baz018 (PMC6379597; doi:10.1093/database/baz018)
Supplement: Supplementary Data [file 20190104-database-supplementary_baz018_supp.docx]

Supplementary Table S1. Description of RNA-seq samples collected in FairBase

| **Genome** | **Editing sites** | **Project** | **Sample ID** | **Sample strain** | **RNA-seq data** | **WGS/SNP** | **Description** | **Editing events** |
| --- | --- | --- | --- | --- | --- | --- | --- | --- |
| *F. graminearum* (PH-1) | 48,508 | PRJNA293594 | SAMN04009308 | PH-1 | SRX1163829 SRX1163861 | - | Perithecia, wild-type, 8 days post-fertilication | 26,056 |
|  |  | PRJNA345628 | SAMN05877051 | Z-3639 | SRX2225609 | SNP | Whole culture, 5 days after sexual induction | 30,937 |
|  |  |  | SAMN05877050 | Z-3639 | SRX2225610 | SNP | Whole culture, Fgdicer1 mutant, 5 days after sexual induction | 28,522 |
|  |  |  | SAMN05877049 | Z-3639 | SRX2225611 | SNP | Whole culture, Fgdicer2 mutant, 5 days after sexual induction | 32,001 |
|  |  |  | SAMN05877048 | Z-3639 | SRX2225612 | SNP | Whole culture, Fgdicer1 Fgdicer2 mutant, 5 days after sexual induction | 31,396 |
|  |  |  | SAMN05877054 | Z-3639 | SRX2225613 | SNP | Whole culture, Fgago1 mutant, 5 days after sexual induction | 29,658 |
|  |  |  | SAMN05877053 | Z-3639 | SRX2225614 | SNP | Whole culture, Fgago2 mutant, 5 days after sexual induction | 25,226 |
|  |  |  | SAMN05877052 | Z-3639 | SRX2225615 | SNP | Whole culture, Fgago1 Fgago2 mutant, 5 days after sexual induction | 30,784 |
|  |  | PRJNA262571 | SAMN03083859 | PH-1 | SRX716770 | - | Perithecia, 96h after induction of sexual development | 5,668 |
|  |  |  | SAMN03083860 | PH-1 | SRX716771 | - | Perithecia, 144h after induction of sexual development | 3,666 |
|  |  | PRJNA376074 | SAMN06349982 | PH-1 | SRX2585917 SRX2585918 | - | Perithecia, amd1 mutant, 7 days post-fertilication | 30,510 |
|  |  |  | SAMN06349977 | PH-1 | SRX2585919 SRX2585920 | - | Perithecia, 7 days post-fertilication | 29,902 |
| F. verticillioides (7600) | 5,227 | PRJNA262571 | SAMN03083853 | Fv999xFv149 | SRX716764 | SRX1649892 | Perithecia; 96h after induction of sexual development | 5,227 |
| *N. crassa*  (FGSC2489) | 47,346 | PRJNA360480 | SAMN06211501 | FGSC17465xFGSC17464 | SRX2493710 SRX2493711 | SRX2493724  SRX2493725 | Perithecia, stc1 mutant, 5 days post-fertilication | 2,450 |
|  |  |  | SAMN06211500 | FGSC11152xFGSC11151 | SRX2493712 SRX2493713 | SRX2493724  SRX2493725 | Perithecia, sad-1 mutant, 5 days post-fertilication | 13,086 |
|  |  |  | SAMN06211499 | FGSC2489xFGSC4200 | SRX2493714 SRX2493715 | SRX2493724  SRX2493725 | Perithecia, 6 days post-fertilication | 35,325 |
|  |  |  | SAMN06211499 | FGSC2489xFGSC4200 | SRX2493716 SRX2493717 | SRX2493724  SRX2493725 | Perithecia, 5 days post-fertilication | 33,579 |
|  |  |  | SAMN06211497 | FGSC2489xFGSC4200 | SRX2493718 SRX2493719 | SRX2493724  SRX2493725 | Perithecia, 4 days post-fertilication | 18,599 |
|  |  |  | SAMN06211496 | FGSC2489xFGSC4200 | SRX2493720 SRX2493721 | SRX2493724  SRX2493725 | Perithecia, 3 days post-fertilication | 2,938 |
|  |  | PRJNA388208 | SAMN07172964 | ISU3972xISU3823 | SRX2865090 | SRX2493724  SRX2493725 | Whole culture, SAD-1(+), 6- day - old perithecial culture | 32,516 |
|  |  |  | SAMN07172965 | ISU3974xISU3824 | SRX2865088 | SRX2493724  SRX2493725 | Whole culture, SAD-1(delta), 6 – day - old perithecial culture | 5,460 |
|  |  | PRJNA217182 | SAMN02334704 | FGSC2489 | SRX339610 | - | Whole culture, rid+, 6-day-old perithecial culture | 5,760 |
|  |  |  | SAMN02334810 | FGSC2489 | SRX339648 | - | Whole culture, rid-, 6-day-old perithecial culture | 10,134 |
|  |  | PRJNA177178 | SAMN01760854 | FGSC2489xFGSC4200 | SRX193461 | SRX2493724  SRX2493725 | Surface perithecia, 120hr after crossing | 1,350 |
| *N. tetrasperma* FGSC2508 | 28,492 | PRJNA257828 | SAMN02979270 | FGSC2508xFGSC2509 | SRX672685 | SRX2493724  SRX2493725 | Perithecia, 72h after crossing | 255 |
|  |  |  | SAMN02979271 | FGSC2508xFGSC2509 | SRX672686 | SRX2493724  SRX2493725 | Perithecia, 96h after crossing | 318 |
|  |  |  | SAMN02979272 | FGSC2508xFGSC2509 | SRX672687 | SRX2493724  SRX2493725 | Perithecia, 120h after crossing | 2,181 |
|  |  |  | SAMN02979273 | FGSC2508xFGSC2509 | SRX672688 | SRX2493724  SRX2493725 | Perithecia, 144h after crossing | 1,159 |
|  |  | PRJNA362742 | SAMN06245890 | FGSC2508xFGSC2509 | SRX2508621 | SRX2493724  SRX2493725 | Perithecia, 6 days post - fertilication | 28,442 |
| *P. omphalodes* CBS100304 | 5,217 | PRJNA177769 | SAMN01766656 | CBS100304 | SRX195961 | - | Sexually developing mycelium | 3,285 |
|  |  |  | SAMN01766657 | CBS100304 | SRX195962 | - | Sexually developing mycelium | 4,953 |
| *S. macrospora* FGSC10222 | 2,423 | PRJNA213728 | SAMN02297726 | FGSC10222 | SRX329201 | - | Total sexual mycelium | 2,423 |

Supplementary Table S2. Comparisons of A-to-I editing databases

| **Database** | **Species** | **Search based on genomic region** | **Search based on sequence** | **Search based on patway** | **Build-in genome browse** | **With editing levels** | **Reference** |
| --- | --- | --- | --- | --- | --- | --- | --- |
| DARNED | 3 | Yes | Yes | No | No | No | (Kiran, et al., 2013) |
| RADAR | 3 | Yes | No | No | No | Not complete | (Ramaswami and Li, 2014) |
| REDIportal | 1 | Yes | No | No | Yes | Yes | (Picardi, et al., 2017) |
| FairBase | 6 | Yes | Yes | Yes | Yes | Yes | This study |
